# Leucine Protects Dry Powders for Inhalation Against Irreversible Moisture-Induced Aggregation

**DOI:** 10.3390/pharmaceutics17111391

**Published:** 2025-10-27

**Authors:** Evalyne M. Jansen, Luke van der Koog, Henderik W. Frijlink, Wouter L. J. Hinrichs

**Affiliations:** 1 Department of Pharmaceutical Technology and Biopharmacy, Groningen Research Institute of Pharmacy, Faculty of Science and Engineering, University of Groningen, 9713 AV Groningen, The Netherlands; e.m.jansen@rug.nl (E.M.J.); h.w.frijlink@rug.nl (H.W.F.); 2 Department of Molecular Pharmacology, Groningen Research Institute of Pharmacy, Faculty of Science and Engineering, University of Groningen, 9700 AD Groningen, The Netherlands; l.van.der.koog@rug.nl; 3 GRIAC, Groningen Research Institute for Asthma and COPD, University Medical Center Groningen, 9700 RB Groningen, The Netherlands

**Keywords:** inulin, leucine, storage stability, spray drying, moisture, LDH, β-gal, vitrification, water replacement

## Abstract

**Background:** Pulmonary administration offers a promising route for certain biologics, particularly when treating respiratory diseases. Spray drying is widely employed to produce inhalable powders with the biologics incorporated in a stabilizing amorphous sugar. Hydrophobic amino acids such as leucine are frequently added to improve dispersibility. **Objectives:** While the aerodynamic benefits of leucine are well established, its influence on irreversible moisture-induced dry powder particle aggregation and protein stability during storage remains less evaluated. **Methods:** In this work, inulin-based powders with and without 4 wt-% leucine were spray dried and stored at 43%, 58%, 69%, and 75% relative humidity (RH) at 20 ± 2 °C. **Results:** Immediately after drying, both formulations displayed comparable physicochemical characteristics. However, during storage of inulin-only formulations, dry powder particles showed viscous flow and formed big irreversible aggregates after storage at an RH of 58% and above, whereas leucine-containing powders remained intact across all tested conditions up to 20 days. Protein stability was assessed using lactate dehydrogenase (LDH) and β-galactosidase (β-gal) as model proteins. At 43% RH, the Tg remained above the storage temperature, and both LDH and β-gal retained their enzymatic activity for up to 20 days. At 75% RH, however, the Tg dropped to below storage temperature, resulting in a loss of stability for LDH, consistent with its reliance on vitrification. In contrast, β-gal maintained its activity at 75% RH, indicative of stabilization through water replacement. **Conclusions:** Overall, these results demonstrate that leucine enhances the physical stability of inulin powders by preventing irreversible aggregation under humid conditions. However, this effect does not extend to protecting proteins reliant on vitrification. These findings highlight the potential of inulin and leucine to reduce the need for stringent storage conditions of biologics.

## 1. Introduction

Pulmonary delivery of biologics represents an effective approach for treating both respiratory and systemic diseases. The pulmonary route offers several therapeutic advantages, including rapid onset of action, high local drug concentrations at the site of disease, and avoidance of first-pass metabolism [1]. Dry powder inhalers (DPIs) are widely used for pulmonary drug administration due to their portability, ease of use, and minimal need for patient coordination during inhalation [2]. Despite these benefits, formulating biologics as dry powder formulations remains challenging. Critical factors such as the physicochemical stability of the biologic in the dry state and the particle size of the powder must be optimized to ensure its suitability for inhalation and therapeutic efficacy.

Spray drying is a widely employed technique to produce inhalable dry powders with favorable aerodynamic properties for pulmonary delivery [3]. In this process, biologics are co-formulated with stabilizing excipients that form an amorphous matrix, thereby preserving structural integrity during both drying and subsequent storage [2,4]. Inulin, a polysaccharide with a relatively high glass transition temperature (Tg), has been investigated as an excipient for its potential to enhance protein stability in spray dried formulations [2]. Compared to disaccharides such as sucrose and trehalose, inulin offers advantages due to its higher Tg, allowing it to remain in the glassy state at high temperatures and relative humidities (RHs), thereby improving its stabilizing capacity [5,6]. However, under storage conditions of high RH, the Tg of inulin may decrease below 20 ± 2 °C, resulting in increased translational molecular mobility and promoting physical instability, including particle fusion through viscous flow. This may negatively impact both protein stability and aerosol performance [7,8,9].

To mitigate moisture-induced challenges related to dispersibility, hydrophobic amino acids such as leucine are frequently incorporated into spray dried formulations. Leucine exhibits moderate surface activity and low aqueous solubility (22 mg/mL at room temperature), enabling its migration toward the droplet surface during spray drying. Upon enrichment at the air–liquid interface, leucine forms a surface layer that reduces interparticle cohesion, thereby improving powder dispersibility and directly enhancing aerodynamic performance [10,11,12]. This aerodynamic benefit is strongly concentration dependent: optimal aerosolization is generally achieved at 2–5% (*w*/*w*) leucine, whereas higher levels (≥10% (*w*/*w*)) may instead compromise aerodynamic properties. At elevated concentrations, leucine can induce a morphological shift from spherical to corrugated dry powder particles and promote the formation of larger surface crystals, resulting in a heterogeneous, non-uniform shell that diminishes aerodynamic properties [1,8,13,14,15,16]. Based on these considerations, 4 wt-% leucine represents an optimal concentration, as it lies within the optimal range for improving dispersibility while maintaining a low excipient load [17,18,19,20]. This is particularly relevant for therapeutic applications, where minimizing excipients is desirable. Moreover, higher leucine concentrations may compromise the stability of co-spray dried proteins, given that leucine is partly hydrophobic whereas proteins are predominantly hydrophilic.

Although leucine’s dispersibility-enhancing effects are well established, its role in preventing moisture-induced destabilization remains less clearly defined. Due to its hydrophobic nature leucine may act as barrier against moisture induced destabilization. Previous research has shown that hydrophobic amino acids, including leucine, can provide moisture protection in spray dried powders [21,22]. For example, trehalose–leucine formulations stored at high RH (>50%) retained favorable aerodynamic properties after storage despite of the transition from its glassy to the rubbery state during storage [16,22,23]. Zhang et al. demonstrated that 5% (*w*/*w*) leucine inhibited trehalose recrystallization at 55% RH [16], whereas Wang et al. reported that leucine was unable to prevent the transition of trehalose to the rubbery state at 90% RH [23]. Similar effects have been observed for small molecule drugs (e.g., disodium cromoglycate and salbutamol) and biologics (e.g., bovine serum albumin) co-formulated with leucine, which exhibited preserved aerosol performance after storage at high RH [8,14,24,25].

Collectively, these findings indicate that leucine can protect dry powders from irreversible moisture-induced aggregation, but it does not prevent a transition from the glassy to the rubbery state of the co-spray dried amorphous excipient at high RH [23]. This limitation is particularly critical for protein stabilization, as protein stability depends on distinct stabilizing mechanisms. Nguyen et al. demonstrated that, when freeze-dried with arginine–pullulan combinations, some proteins are mainly protected through water replacement, whereas others rely more strongly on vitrification [26]. Whether these mechanisms operate similarly in spray dried proteins with inulin, with or without leucine, remains unclear. In the water replacement mechanism, sugar molecules substitute for water molecules by forming hydrogen bonds with the protein, thereby maintaining structural integrity of the protein even under plasticized conditions [4,16,27]. In contrast, vitrification stabilizes proteins by embedding them in a matrix in the glassy state that kinetically immobilizes molecular motion. Under high RH conditions, however, moisture uptake induces plasticization, lowering the Tg below the storage temperature. This reduction in Tg increases translational molecular mobility, and stabilization of the biologic by vitrification is lost [27]. Because leucine cannot prevent water uptake and subsequent plasticization, proteins of which the stabilization relies on vitrification, such as lactate dehydrogenase (LDH), may lose stability once the Tg is surpassed. Therefore, it remains unclear whether proteins like LDH can be effectively stabilized when co-formulated with a sugar and leucine stored under high-humidity conditions.

To the best of our knowledge, it was neither investigated whether leucine can protect inulin-based spray dried formulations from irreversible particle aggregation, nor has this been related to the stability of encapsulated proteins that rely on stabilization mechanisms (i.e., vitrification and/or water replacement). In this work, we address this gap by systematically evaluating the effect of leucine on inulin-based powders under controlled RH conditions (43%, 58%, 69%, and 75%) at 20 ± 2 °C. First, the physicochemical properties and primary particle sizes were examined to assess the influence of leucine on the moisture sensitivity of inulin. In parallel, the stabilizing capacity of inulin, with and without leucine, was investigated using two model enzymes with distinct stabilization mechanisms: lactate dehydrogenase (LDH) and β-galactosidase (β-gal). Together, this study provides a novel framework for assessing the role of leucine in mitigating irreversible moisture-induced aggregation in spray dried inulin-based formulations and clarifies its relevance for protein stabilization under high humidity.

## 2. Materials and Methods

### 2.1. Materials

Inulin (Frutafit TEX, molecular weight 4 kDa) was generously provided by Sensus (Roosendaal, the Netherlands). L-leucine was purchased from Ofipharma (Ter Apel, The Netherlands) (1711027.1653). HEPES(H4034-100G) was acquired from Sigma Aldrich (St. Louis, MO, USA). Sodium dihydrogen phosphate (1.06342.1000), potassium dihydrogen phosphate (1.04873.1000), disodium hydrogen phosphate (1.06580.1000), O-nitrophenyl-β-D-galactopyranoside (ONPG) (4151737) and lactate dehydrogenase, rabbit muscle (427217-25KU) were obtained from Merck (Darmstadt, Germany). Magnesium chloride hexahydrate (63033) and bovine serum albumin (BSA) (A3912-50G) were obtained from Sigma Aldrich (St. Louis, MO, USA). β-galactosidase (GAH-201, 8176409000) obtained from Sorachim (Lausanne, Switzerland). Potassium carbonate (209619-500G), strontium chloride, and sodium bromide (310506-500G) were purchased from Sigma Aldrich (St. Louis, MO, USA). Sodium chloride (76051275-1000) was purchased from Boom (Meppel, the Netherlands). NADH (39267135) was purchased from Roche diagnostics (Basel, Switzerland). Sodium pyruvate(P2256-5G). was obtained from Sigma Aldrich (St. Louis, MO, USA). 

### 2.2. Formulations for Spray Drying

Two placebo formulations were made: (1) inulin (2.5 *w*/*v*) and (2) inulin (2.5% *w*/*v*) supplemented with 4 wt-% leucine. Furthermore, four protein formulations were made: (3) inulin (2.5% *w*/*v*) with β-gal (0.03% *w*/*v*), (4) inulin (2.5% *w*/*v*), 4 wt-% leucine and β-gal (0.03% *w*/*v*), (5) inulin (2.5% *w*/*v*) with LDH (0.03% *w*/*v*) and (6) inulin (2.5% *w*/*v*), 4 wt-% leucine and LDH (0.03% *w*/*v*) (Table 1). All formulations were prepared in 20 mM HEPES buffer (pH 7.4). A weight ratio of 1:99 protein (β-gal or LDH) to total solute content (inulin, leucin, and HEPES) was used.

### 2.3. Spray Drying

Spray drying was carried out using a Büchi B-290 system combined with a B-296 dehumidifier. A feed rate of 1 mL/min was applied using an NE-300 syringe pump (ProSense B.V., Oosterhout, The Netherlands) connected to a 60 mL syringe (Codan B.V., Deventer, The Netherlands). The formulation was transferred from the syringe to the nozzle (1.5 mm cap) through rubber tubing. Atomizing air was set to 601 Ln/h (rotameter at 50 mm), with an inlet temperature of 60 °C and aspirator operating at full capacity (100%). These conditions yielded an outlet temperature of 36 °C. Powder was collected in a collection vial (20 mL EPA vial, Thermo Scientific, Waltham, MA, USA). The powder yield, typically above 80%, was determined gravimetrically by comparing the weight of the powder collection vessel mass before and after drying. Obtained powders were either analyzed directly or stored at room temperature (20 ± 2 °C) and 43%, 58%, 69%, or 75% RH without lid for 2 h, 6 h, 1 day, 5 days, and 20 days. A 43% RH environment was established by placing a saturated potassium carbonate solution in a desiccator. A 58% RH environment was established by placing a saturated sodium bromide solution in a desiccator. A 69% RH environment was established by placing saturated strontium chloride solution in a desiccator. A 75% RH environment was established by placing saturated sodium chloride solution in a desiccator. Each formulation was prepared as a single batch to ensure that all subsequent tests were conducted on material from the same production lot.

### 2.4. Differential Scanning Calorimetry

The Tg of spray dried inulin powders, both with and without 4 wt-% leucine (formulations 1 and 2), was determined after spray drying by differential scanning calorimetry (DSC) using a Q2000 calorimeter from TA Instruments (New Castle, DE, USA). About 5–10 mg of spray dried powder was loaded in a T-zero pan without a lid. Each sample was first heated to 90 °C at a rate of 20 °C per minute and then held isothermal for 15 min to ensure complete water evaporation. Consequently, the samples were cooled at a rate of 20 °C per minute to −90 °C. Lastly, the samples were heated again to 180 °C at a rate of 20 °C per minute. The Tg was identified by finding the inflection point in the reversing heat flow versus temperature in the thermogram. Spray dried inulin with and without 4 wt-% leucine was stored in open glass vessels for 1 day under controlled conditions of 43%, 58%, 69%, and 75% RH and 20 ± 2 °C. Following storage, the Tg was determined using DSC with a heat–cool–heat run in closed aluminum T-zero pans. Initially, samples were cooled to −90 °C and held for 10 min. Subsequently, they were heated to 90 °C at a rate of 20 °C per minute. This thermal cycle was repeated by cooling the samples again to −90 °C, followed by reheating to 90 °C, both at a heating rate of 20 °C per minute. The Tg was identified by locating the inflection point on the first heat flow versus temperature curve. All analyses were conducted in triplicate to ensure reproducibility.

### 2.5. X-Ray Powder Diffraction

X-ray powder diffraction (XRPD) was carried out using a Bruker D2 Phaser (Billerica, MA, USA) on placebo formulations of spray dried inulin, both with and without 4 wt-% leucine (formulations 1 and 2), directly upon spray drying. Measurements were taken across a 2θ range of 5–60°, with steps of 0.05° and a duration of 1 s per step. The detector aperture was fixed at 5°, and the stage rotated at 15 revolutions per minute during data collection. A 1 mm divergence slit and a 3 mm air scatter screen were applied. Samples were placed in a Si low-background holder. Formulations 1 and 2 were measured once directly after spray drying.

### 2.6. Dynamic Vapor Sorption

The water sorption behavior of spray dried inulin or inulin with 4 wt-% leucine (formulations 1 and 2) were assessed using a dynamic vapor sorption (DVS)-1000 gravimetric analyzer (Surface Measurement Systems Ltd., London, UK). Samples weighing about 10 mg were tested at 25 °C under ambient pressure. Moisture uptake by the inulin and inulin with 4 wt-% leucine formulation were monitored over a relative humidity (RH) range of 0% to 90%, increasing in 10% steps where the RH was raised only after mass equilibrium was reached, defined as a change below 0.5 μg over a 10 min interval. Furthermore, moisture uptake of the inulin and inulin with 4 wt-% leucine formulations was monitored at set RHs (43%, 58%, 69%, and 75%). The measurement was considered finished when an equilibrium was reached at 600 min.

### 2.7. Thermogravimetric Analysis

Thermogravimetric analysis was performed to determine the moisture content of spray dried inulin formulations, with and without 4 wt-% leucine (formulations 1 and 2). Samples were stored for 1 week at controlled conditions of 43%, 58%, 69%, and 75% RH and 20 ± 2 °C without lid. TGA was conducted in duplicate by heating the samples from room temperature to 95 °C at a rate of 10 °C per minute. Weight loss up to 95 °C was used as a measure of the water content in the samples.

### 2.8. Primary Particle Size Analysis

The geometric size distribution of spray dried inulin and inulin with 4 wt-% leucine (formulations 1 and 2) were assessed in triplicate by laser diffraction. A RODOS dry powder disperser (Sympatec, Clausthal-Zellerfeld, Germany), operating at 3 bar, was used in combination with a HELOS BF laser diffractometer (Sympatec, Clausthal-Zellerfeld, Germany) fitted with an R3 lens (100 mm). About 10 mg of powder was placed on a spinning disk, and the measurement started once optical density reached 0.2% on channel 30. Each scan lasted 3 s. Particle size parameters (X50) were determined based on Fraunhofer diffraction theory.

### 2.9. Scanning Electron Microscopy

Particle morphology of spray dried inulin and inulin with 4 wt-% leucine (formulations 1 and 2) were examined by scanning electron microscopy (SEM). Spray dried inulin and inulin 4 wt-% leucine were analyzed by SEM directly upon spray drying and after storage for 1 day at 0%, 43%, 58%, 69%, and 75% RH and 20 ± 2 °C without lid. A JSM 6460 microscope (Jeol, Tokyo, Japan) was used. Powder samples were mounted onto aluminum stubs with double-sided carbon tape. Before imaging, a gold/palladium layer (~10 nm) was applied by sputter-coating using a JFC-1300 auto fine coater (Jeol, Tokyo, Japan). Images were acquired under vacuum at 10 kV acceleration voltage, a 10 mm working distance, and a spot size of 25.

### 2.10. Storage Stability of Spray Dried Model Proteins

To evaluate storage stability, spray dried inulin with β-gal or LDH and inulin with 4 wt-% leucine with β-gal or LDH (formulations 3–6) were stored at 43% or 75% RH and a temperature of 20 ± 2 °C. Samples were stored at these controlled conditions without lid for 5 days and 20 days. The enzymatic activity of two model enzymes, β-gal and LDH, was measured to assess their stability, following the method described by Tonnis et al. [28]. For β-gal, activity was quantified using a kinetic assay based on the conversion of ortho-nitrophenyl-β-galactoside (ONPG) (colorless) to the chromogenic product ortho-nitrophenol (yellow). LDH activity was determined by its ability to convert pyruvate to lactate. Enzyme stability was expressed as the percentage of remaining activity relative to the theoretical value derived from a calibration curve.

### 2.11. Statistics

Results are shown as mean with standard deviation (SD), and both sample size and number of replicates are indicated in the relevant figure captions. For evaluating differences across multiple groups, a two-way ANOVA was employed, followed by Dunnett’s post hoc test for multiple comparisons. A *p*-value below 0.05 was considered statistically meaningful. All analyses were carried out using GraphPad Prism software, version 10 (GraphPad Software).

## 3. Results

### 3.1. Dry Powder Characteristics of Spray Dried Inulin and Inulin–Leucine Formulations

**Figure 1:**
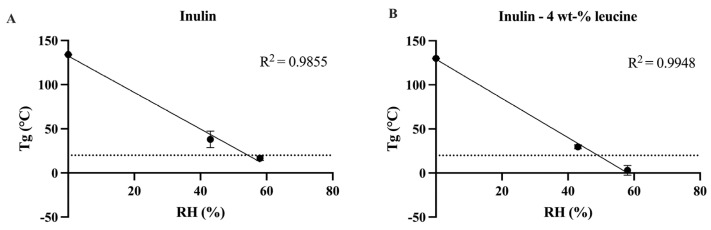
Linear regression between the relative humidity (RH) and Tg of spray dried inulin and inulin 4 wt-% leucine. (**A**) Tg of spray dried inulin directly upon spray drying, after storage for 1 day at 43% RH and after storage for 1 day at 58% RH, line shown at 20 °C. (**B**) Tg of spray dried inulin formulated with 4 wt-% leucine directly upon spray drying, after storage for 1 day at 43% RH and after storage for 1 day at 58% RH, line shown at 20 °C. Relative humidity (RH) shown in percentage (%). *N* = 3, mean ± SD.

Initially, the physicochemical characteristics of spray dried inulin, both with and without the addition of 4 wt-% leucine, were evaluated to elucidate the influence of leucine incorporation. DSC measurements conducted immediately post spray drying indicated a Tg of 134.01 ± 0.29 °C for the inulin formulation without leucine. The Tg of the formulation without leucine was marginally, yet significantly, higher than that of the leucine-containing formulation (130.13 ± 0.37 °C) (Figure 1, Figures S1 and S2). This reduction was unexpected, as the addition of a small amount of leucine (4 wt-%) was not anticipated to reduce the Tg of the spray dried matrix. Subsequently, the Tg of spray dried inulin was examined after 1 day of storage under controlled RH conditions of 43% and 58% at 20 ± 2 °C. After storage at 43% RH, a distinct Tg was observed at 42.41 ± 1.54 (Figure 1 and Figure S1). At 58% RH, the Tg remained detectable. However, the glass transition became broader and less sharply defined at 16.70 ± 2.15 (Figure 1 and Figure S1). At higher humidities (69% and 75% RH), the spray dried inulin samples exhibited viscous flow, which prevented further DSC analysis due to handling problems (Figure S3). Nevertheless, the Tg of spray dried inulin stored at 69% and 75% RH can be expected to fall below room temperature (20 ± 2 °C), as inferred from the Tg values obtained for samples stored at 0% to 58% RH. Comparable trends were noted for spray dried inulin containing 4 wt-% leucine. Following storage at 43% RH and 20 ± 2 °C, a distinct Tg was recorded at 29.82 ± 1.89 °C. At 58% RH and 20 ± 2 °C, although the Tg broadened and became less distinct, it remained observable at 3.41 ± 4.01 °C (Figure 1 and Figure S2). In contrast, at 69% and 75% RH and 20 ± 2 °C, the Tg was too diffuse to be reliably identified (Figure S2), but based on the lower RH data, these Tg values lay below room temperature (20 ± 2 °C). The results indicate that exposure to high humidity leads to plasticization of the inulin matrix, thereby reducing its Tg.

**Figure 2:**
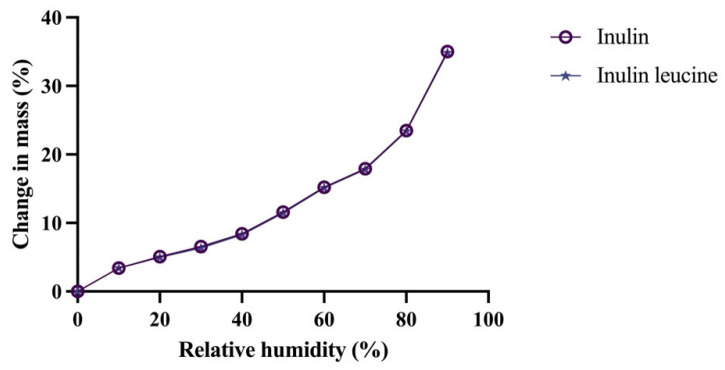
Moisture uptake of spray dried inulin and inulin with 4 wt-% leucine is similar. Data shown as change in mass in percentage (%), relative humidity shown in percentage (%).

DVS analysis was subsequently conducted to investigate the moisture uptake profiles of both formulations. Comparable moisture sorption behavior of spray dried inulin and spray dried inulin with leucine was observed, indicating that the addition of leucine did not significantly alter the hygroscopicity of spray dried inulin (Figure 2). No recrystallization events were detected during the DVS measurements (Figure 2). Moreover, overall moisture uptake increased proportionally with RH and remained similar for both formulations (Figure S4). TGA experiments supported these findings, demonstrating increased weight loss at higher RH, without significant differences between the two formulations (Figure S5). In addition, XRPD analysis confirmed the amorphous nature of spray dried inulin and inulin with 4 wt-% leucine formulations immediately after spray drying, as evidenced by the absence of distinct Bragg peaks (Figure S6). Although leucine is known to crystallize upon spray drying, the concentration used (4 wt-%) was too low to detect crystalline domains by XRPD [8,14]. In summary, these results demonstrate that incorporating 4 wt-% leucine did not significantly affect the moisture sorption behavior or solid-state characteristics of spray dried inulin powders. Interestingly, despite these similarities, the Tg of the leucine-containing formulation was significantly lower than that of pure inulin, which is a surprising finding given the low leucine concentration.

### 3.2. Leucine Protects Spray Dried Inulin Against Irreversible Moisture-Induced Aggregation

**Figure 3:**
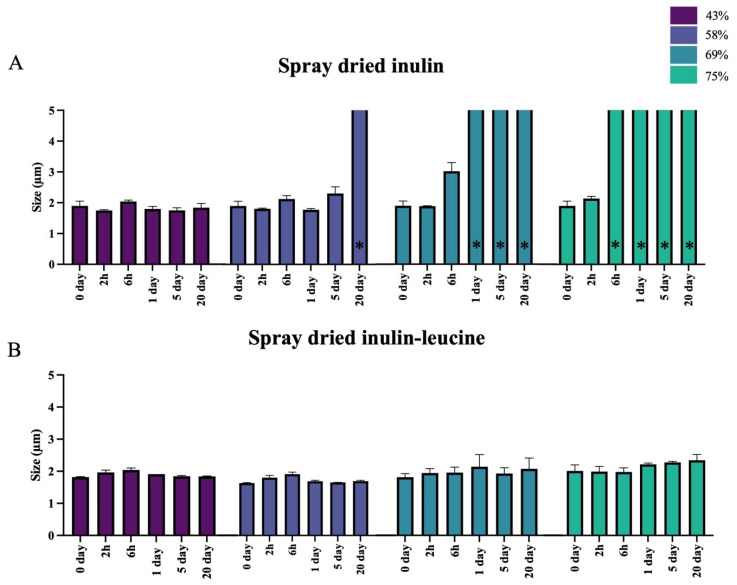
The addition of leucine ensures that spray dried inulin maintains its primary particle size when stored at different RHs. (**A**) Size (X50) of spray dried inulin. Samples were stored at 43%, 58%, 69%, and 75% relative humidity (RH) and 20 ± 2 °C for 2h, 6h, 1 day, 5 days, and 20 days. (**B**) Size (X50) of spray dried inulin with 4 wt-% leucine. Samples were stored at 43%, 58%, 69%, and 75% relative humidity (RH) and 20 ± 2 °C for 2h, 6h, 1 day, 5 days, and 20 days. * = samples that exhibited viscous flow could not be measured using laser diffraction. Severely aggregated particles are indicated by bars that pass the upper axis, *N* = 3, mean ± SD.

To evaluate the protective effect of leucine against irreversible moisture-induced aggregation, inulin was spray dried with and without 4 wt-% leucine and stored at 43%, 58%, 69%, and 75% RH at 20 ± 2 °C. Immediately upon spray drying, the primary particle size of the spray dried inulin and inulin–leucine formulation were comparable, showing no significant differences (Figure 3A,B). This indicates that leucine does not influence the particle size of spray dried inulin. The primary particle size was examined upon storage for 2 h, 6 h, 1 day, 5 days, and 20 days. Inulin-only powders displayed a clear humidity- and time-dependent aggregation, accompanied by the onset of viscous flow at elevated RH (Figure 3A and Figure S3). Specifically, at 58% RH, viscous flow developed only after 20 days; at 69% RH, it already occurred within day 1; and at 75% RH, it was evident within just 6 h, preventing laser diffraction measurements in all cases. When viscous flow occurred, the dry powder particles could no longer be measured using laser diffraction. It was therefore assumed that the particles exceeded 5 µm in size. Consequently, particle sizes > 5 µm are shown in Figure 3A to indicate irreversible particle aggregation. In contrast, the primary particle size of leucine-containing formulations remained unchanged for up to 20 days across all RH conditions (Figure 3B). These results demonstrate that leucine effectively prevents the severe agglomeration observed in the leucine-free formulations, which could not be dispersed even under the high dispersion forces generated by the RODOS dry powder disperser operating at 3 bar.

Differences between the inulin and inulin–leucine formulations were also evaluated. Only the dry powder particles that did not exhibit aggregation due to viscous flow were included in this analysis. At 43% and 58% RHs, a small but significant difference in particle size was observed between spray dried inulin and inulin–leucine after 2 h of storage. At 58% RH, this difference remained significant after 1 and 5 days of storage. Furthermore, at 69% RH, a significant difference in particle size was already evident after 6 h of storage. At 75% RH, this analysis could not be performed, since the dry powder particles of the inulin formulation irreversibly aggregated due to viscous flow.

**Figure 4:**
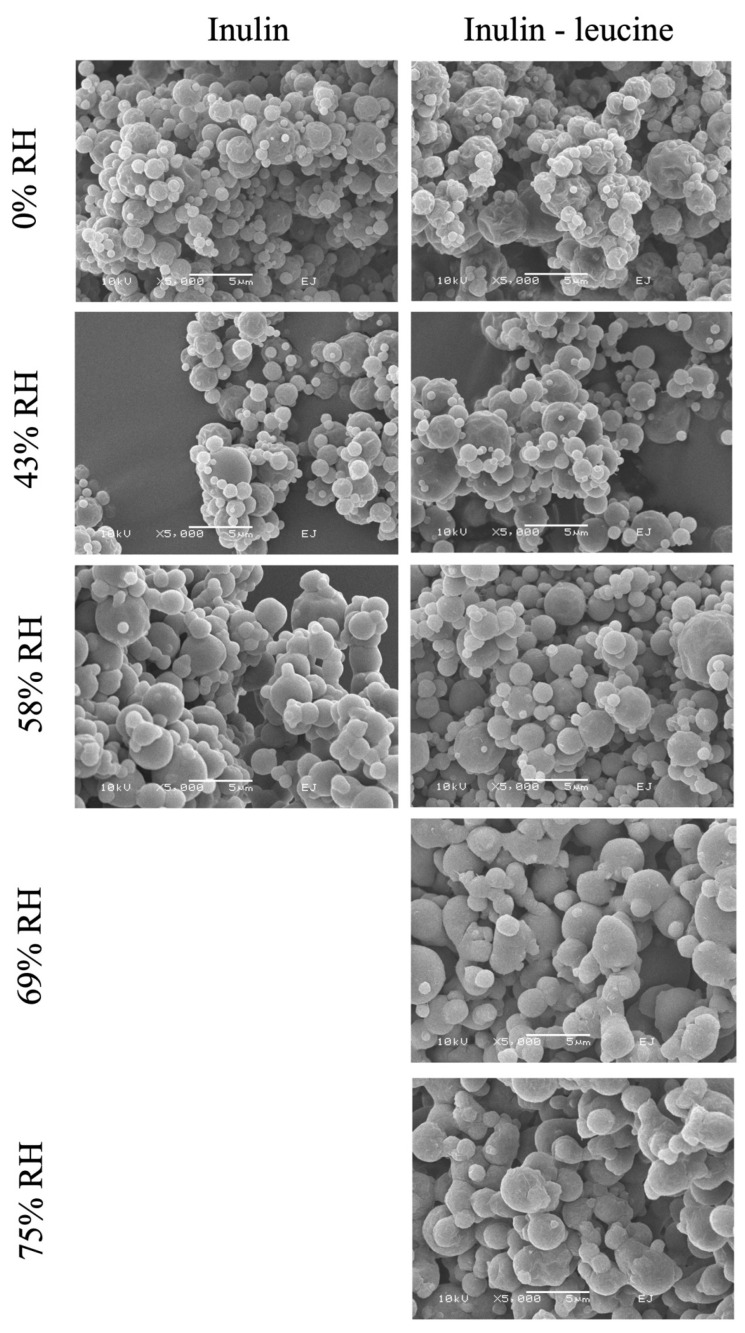
SEM images of spray dried inulin and inulin–leucine stored at 0%, 43%, 58%, 69%, and 75% RH at 20 ± 2 °C. 5000× magnification. Spray dried inulin stored at 69% and 75% RHs showed viscous flow so visualization with SEM was not possible.

SEM images showed that the spray dried particles of both formulations have a spherical somewhat raisin-like structure (Figure 4). Satellite particles adhere to the spray dried leucine. For spray dried inulin stored at 69% and 75% RH and 20 ± 2 °C no SEM picture could be taken, since these particles completely fused by viscous flow (Figure S3). Overall, these findings demonstrate that leucine effectively protects spray dried inulin powders from moisture-induced irreversible aggregation. In the absence of leucine, the extent and speed of irreversible aggregation increased with increasing RH, highlighting the importance of leucine as a moisture-protective excipient for maintaining the physical stability of spray dried dry powder formulations during storage.

### 3.3. Protein Activity Is Not Influenced by the Addition of Leucine

Leucine protects spray dried inulin particles from irreversible moisture-induced aggregation, thereby enhancing their physical stability at high RH. While leucine preserves the external particle morphology, the amorphous inulin core remains susceptible to water-induced plasticization. Plasticization lowers the Tg, and once the Tg drops below room temperature, the capacity of inulin to stabilize proteins through vitrification is compromised. To further investigate this issue, we investigated whether the incorporation of leucine affects the stability of proteins that rely on either vitrification or water replacement as their primary stabilization mechanism. Two model proteins with distinct stabilization mechanisms were selected: LDH and β-gal. The formulations were stored at 43% RH, where the Tg was above storage temperature (20 ± 2 °C), and at 75% RH, where the Tg falls below the storage temperature (20 ± 2 °C). Enzymatic assays were employed as protein stability indicating technique.

**Figure 5:**
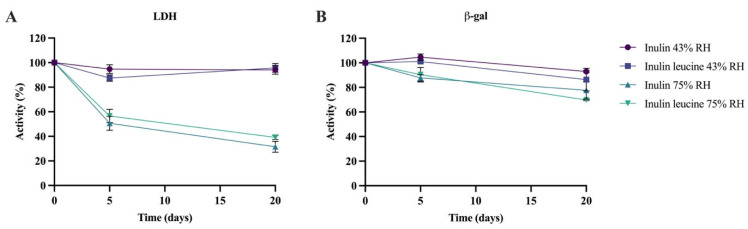
LDH and β-gal activity after spray drying with inulin and inulin–leucine and after storage at 43% and 75% RH and 20 ± 2 °C. (**A**) LDH spray dried with inulin and inulin–leucine and stored for 5 and 20 days at 43% and 75% RH and 20 ± 2 °C. (**B**) β-gal spray dried with inulin and inulin–leucine and stored for 5 and 20 days at 43% and 75% RH and 20 ± 2 °C. Data are normalized to the activity directly after spray drying. Mean ± standard deviation (SD), RH = relative humidity, *n* = 3.

LDH and β-gal were spray dried with either inulin alone or inulin containing 4 wt-% leucine and subsequently stored at 43% and 75% RH at 20 ± 2 °C for 5 days and 20 days. Immediately after spray drying, enzymatic activity remained above 86% for all formulations. To facilitate comparison, all subsequent storage stability data were normalized to the initial activity values. For LDH, activity was well maintained at 43% RH throughout the 20-day storage period, and no notable differences were observed between inulin-only and inulin–leucine formulations (Figure 5A). At 75% RH, however, the enzymatic activity of LDH decreased significantly over time. In the inulin-only formulation, the enzymatic activity dropped to 50.6 ± 5.7% after 5 days and further declined to 31.5 ± 4.4% after 20 days. A similar pattern was seen for the inulin–leucine formulation, with an enzymatic activity reduced to 56.5 ± 5.4% after 5 days and to 39.1 ± 1.8% after 20 days of storage (Figure 5A).

Similarly to LDH, β-gal remained stable at 43% RH and 20 ± 2 °C, up to 20 days of storage when spray dried with inulin alone (Figure 5B). For the inulin–leucine formulation, the enzymatic activity was unchanged after 5 days, but a small yet significant decline was observed after 20 days. Nevertheless, 86.4 ± 9.2% of β-gal activity was retained after 20 days of storage at 43% RH of the inulin–leucine formulation. At 75% RH and 20 ± 2 °C, both the inulin and inulin–leucine formulations showed a small but significant decline in β-gal activity after 5 and 20 days of storage. After 5 days, 87.8 ± 2.6% and 90.4 ± 6.0% of the initial activity were retained for the inulin and inulin–leucine formulations, respectively. After 20 days, 77.7 ± 7.1% and 69.7% ± 0.5% of the enzymatic activity were maintained for the inulin and inulin–leucine formulations, respectively. All in all, for β-gal the enzymatic activity was largely preserved across both humidity levels and formulations, with only modest activity reductions during storage.

Collectively, these findings demonstrate that leucine does not inhibit moisture uptake in the amorphous inulin core. At 43% RH and 20 ± 2 °C, where the Tg remains above the storage temperature, inulin retains its amorphous state and protein stabilization via vitrification is preserved, independent of leucine addition. In contrast, at 75% RH and 20 ± 2 °C the Tg falls below the storage temperature, resulting in the loss of vitrification, a process that leucine cannot prevent. Consequently, only biologics that do not depend on vitrification for stabilization can remain enzymatically stable under humid storage conditions when spray dried with inulin, irrespective of the presence of leucine.

## 4. Discussion

In this study, we investigated the role of leucine in mitigating irreversible moisture-induced aggregation in spray dried inulin particles and evaluated the influence of leucine on the protein stability enhancing effect of inulin using the model proteins LDH and β-gal. Initially, the physicochemical properties of the spray dried inulin and inulin–leucine formulations were characterized. Interestingly, both formulations exhibited comparable physicochemical properties. Particle size analysis, DVS, and TGA revealed similar size and moisture uptake profiles, also XRPD patterns showed no significant differences between the two formulations. This observation is noteworthy given that previous studies have reported that the incorporation of leucine often influences the water sorption behavior and crystallinity of spray dried powders. However, those studies typically employed higher leucine concentrations, frequently 20% (*w*/*w*) or more. For example, with spray dried formulations of trehalose, salbutamol, or disodium cromoglycate with varying leucine concentrations it was shown that increasing the leucine content reduced water uptake and increased the overall crystalline fraction in the spray dried formulations [8,14,22]. This effect is consistent with leucine’s hydrophobic nature, which limits water absorption. In contrast, Li et al. investigated the effects of lower leucine concentrations (2 wt-% and 5 wt-%) resembling the leucine concentration used in this study (4 wt-%). They found, similar to our results, that at such low levels, leucine did not significantly alter moisture uptake or the crystallinity of the spray dried powders [8,14]. These findings collectively suggest that leucine, when used at low concentrations, does not markedly affect the physicochemical properties of spray dried amorphous formulations.

Despite the limited influence of leucine on the physicochemical properties of spray dried inulin, leucine provided clear protection against irreversible moisture-induced aggregation. The inulin–leucine formulation remained physically stable and did not exhibit irreversible aggregation when stored at 43%, 58%, 69%, or 75% RH and 20 ± 2 °C for up to 20 days. The inulin-only formulation underwent irreversibly aggregation at higher RH levels. This protective effect of leucine is particularly relevant because the amorphous inulin core remained highly susceptible to water-induced plasticization even in the presence of leucine. Water is a well-known plasticizer for amorphous materials and can significantly reduce the Tg, even at low levels of moisture uptake [29]. Disaccharides such as sucrose and trehalose are particularly sensitive to this effect. The Tg of sucrose and trehalose decreases to below 20 ± 2 °C even at relatively low RH, limiting their ability to stabilize biologics stored under humid conditions [6]. Inulin, in contrast, has a substantially higher Tg, enabling it to remain in the glassy state at moderate RH and 20 ± 2 °C. Nevertheless, previous studies demonstrated that the Tg of amorphous inulin also decreases progressively with increasing RH [9]. Our findings confirm this behavior: the Tg of spray dried inulin with or without leucine decreased notably when stored at 43% and 58% RH at 20 ± 2 °C. At higher RH levels (69% and 75%), the Tg of inulin–leucine formulations could not be reliably determined, as water plasticization resulted in broad, poorly defined transitions. Moreover, the inulin-only formulation showed viscous flow within one day of storage at 69% and 75% RH levels and 20 ± 2 °C, preventing DSC measurements altogether. These observations align with the work of Ronkart et al., who showed that inulin has a high Tg and can withstand moisture to a high extent, it can still undergo physical instability under high humid conditions [30].

The protective effect of leucine against irreversible, moisture-induced particle aggregation can be attributed to its tendency to migrate toward and accumulate at the particle surface during the spray drying process. Previous studies have clearly demonstrated that leucine preferentially enriches at the particle–air interface, forming a surface coating that serves as a physical barrier and enhances powder dispersibility and physical stability [8,11,13,17,31]. Laser diffraction analysis confirmed that the addition of 4 wt-% leucine to spray dried inulin preserved the primary particle size after storage at RHs up to 75% and a temperature of 20 ± 2 °C for up to 20 days, indicating reduced irreversible moisture-induced aggregation and improved physical stability of the powder. When co-formulated with inulin, leucine orients most likely preferentially at the air–liquid interface. This is due to its amphiphilic structure, with hydrophilic amino and carboxyl groups and a hydrophobic isobutyl side chain [22,32]. During spray drying, the hydrophilic moieties orient toward the aqueous droplet interior, whereas the hydrophobic isobutyl side chain aligns outwards toward the air interface. Rapid water evaporation drives leucine accumulation at this interface, where its local concentration exceeds its solubility limit and leads to precipitation [11]. In contrast, the more soluble inulin remains uniformly distributed within the droplet core. This interfacial alignment of leucine promotes the formation of a leucine-enriched shell that acts as a moisture barrier after drying. Collectively, this explains leucine’s well-established role as a dispersibility enhancer and moisture protector in spray dried formulations [10,11].

For pulmonary delivery, spray dried formulations could be administered using a DPI. DPIs have many advantages over other inhalation devices, such as metered dose inhalers (MDIs) and nebulizers, since DPIs improve stability, dose consistency, portability, and have a reduced environmental impact due to a lower carbon footprint [33,34]. The Cyclops DPI could be a suitable device for the inhalation of the inulin–leucine formulation used in this study, as the Cyclops DPI operates based on a strong dispersion principle. This enables efficient deagglomeration of the powder during inhalation. Previous studies have shown that when the primary particle size of the formulation is within the optimal range for deep lung deposition (1–5 µm), the emitted particle size upon inhalation with the Cyclops DPI is also expected to be appropriate for effective lung deposition [17,35]. However, factors such as residual water content during storage may influence the aerosol performance of spray dried powders. In the present study, the aerosolization behavior of spray dried inulin and inulin–leucine formulations after storage at high RH was not evaluated. Therefore, future studies could evaluate how storage-related changes in moisture content affect the aerosolization efficiency and in vitro deposition behavior of these formulations using cascade impaction analysis.

While leucine effectively limits irreversible moisture-induced aggregation, it does not prevent plasticization of the amorphous inulin core. To assess whether spray dried inulin–leucine dry powder formulations could still confer protection to an encapsulated protein at high humidity, protein stability of two model proteins with different stabilization mechanisms was investigated. At 43% RH, where the Tg of the inulin matrix was above the storage temperature of 20 ± 2 °C and thus in the glassy state, both LDH and β-gal remained stable. At 75% RH, however, the Tg dropped below storage temperature (20 ± 2 °C), leading to a glass to rubber transition of the inulin core and in an increased translational molecular mobility. This resulted in a pronounced loss of LDH activity. Demonstrating that vitrification is essential for LDH stabilization. In contrast, β-gal retained activity even at 75% RH and 20 ± 2 °C. Although the Tg of inulin dropped below 20 ± 2 °C at 75% RH, increasing molecular mobility, β-gal remained stable. This behavior is consistent with the water replacement mechanism, where hydroxyl groups of the sugar matrix form hydrogen bonds with the protein during drying, preserving the native structure of β-gal independently of the Tg [27]. These findings agree with Nguyen et al., who investigated the stabilization of LDH and β-gal upon freeze drying using arginine–pullulan combinations [26]. They demonstrated that LDH is more dependent on vitrification than β-gal, as LDH degradation accelerates upon moisture uptake by the amorphous matrix, which increases molecular mobility and thereby compromises stabilization through vitrification. In contrast, β-gal activity was hardly affected by moisture uptake, indicating a greater reliance on water replacement [26]. In our study, which employed spray drying to produce LDH and β-gal powders using inulin with or without leucin, a similar distinction between the two proteins was observed. Taken together, the results of Nguyen et al. and our study demonstrate that these fundamental stabilization mechanisms (vitrification and water replacement) are universally applicable across different drying techniques, including both freeze drying and spray drying, and with various excipient systems [26]. Furthermore, our results indicate that while leucine effectively reduces irreversible, moisture-induced particle aggregation and preserves the physical integrity of spray dried powders, it does not prevent degradation of proteins that depend primarily on vitrification for stabilization.

Overall, our study highlights the importance of tailoring stabilization strategies to the specific needs of individual proteins in spray dried formulations. As different proteins rely on distinct mechanisms for maintaining stability, such as vitrification or water replacement, a one-size-fits-all approach is unlikely to be effective. Future work should focus on systematically investigating combinations of sugars and amino acids to optimize both physical and biochemical stability across a range of environmental conditions. In this context, leucine emerges as a promising excipient due to its dual role in enhancing powder flowability and protecting against irreversible moisture-induced aggregation, particularly relevant for formulations intended for use in hot and humid climates. To further refine stabilization approaches, the application of Design of Experiments (DoE) could provide a structured framework for identifying optimal excipient compositions and storage conditions tailored to specific protein profiles. Future studies should also include longer-term stability assessments (e.g., 3–6 months) to fully qualify the formulations for industrial application. This strategy would support the development of robust protein therapeutics with improved shelf-life and global applicability.

## 5. Conclusions

In this study, we showed that the presence of 4 wt-% leucine did not substantially alter the physicochemical properties of spray dried inulin, but it does effectively prevent moisture-induced irreversible aggregation of spray dried inulin dry powder particles. However, leucine does not inhibit moisture uptake within the amorphous inulin core at high humidity (>58% RH). As a result, proteins that rely on vitrification by a sugar glass for stability, such as LDH, cannot be effectively stabilized by inulin and leucine under high RH. In contrast, proteins such as β-gal, stabilized via water replacement mechanisms, retain their activity when spray dried with inulin with or without leucine, even when stored above the Tg. This study highlights that leucine primarily protects dry powder particles from humidity-driven physical destabilization, rather than directly stabilizing proteins. This mechanistic insight challenges the prevailing view of leucine as a general protein stabilizer and instead highlights its unique role in safeguarding powder integrity under ambient conditions. By reducing irreversible moisture-induced aggregation at normal RH levels, leucine enables spray dried inulin-based powders to be stored and handled outside of (ultra-)dry environments. This positions leucine as a key excipient for developing stable, inhalable formulations that are easier to distribute and more practical to use globally, particularly in resource-limited settings.

## Figures and Tables

**Table 1:** Tg of spray dried inulin and inulin with 4 wt-% leucine.

Formulation	Sugar	Leucine	Protein
1	Inulin		
2	Inulin	Leucine	
3	Inulin		β-gal
4	Inulin	Leucine	β-gal
5	Inulin		LDH
6	Inulin	Leucine	LDH

β-gal: β-galactosidase; LDH: lactate dehydrogenase.

## Data Availability

The raw data supporting the conclusions of this article will be made available by the authors on request.

## Supplementary Materials

The following supporting information can be downloaded at https://www.mdpi.com/article/10.3390/pharmaceutics17111391/s1; Figure S1. Typical example of DSC measurements of spray dried inulin. Figure S2. Typical example of DSC measurements of spray dried inulin with 4 wt-% leucine. Figure S3. Spray dried inulin and inulin–leucine stored at 43%, 58%, 69%, and 75% RH for 1 day. Figure S4. DVS measurement of spray dried inulin and spray dried inulin with 4 wt-% leucine. Figure S5. TGA measurement of spray dried inulin and spray dried inulin with 4 wt-% leucine. Figure S6. XRPD analysis showed the amorphous structure of spray dried inulin and inulin-leucine formulations.

## Abbreviations

β-gal	β-Galactosidase
DPI	Dry powder inhaler
DVS	Dynamic vapor sorption
FPF	Fine particle fraction
LDH	Lactate dehydrogenase
LD	Laser diffraction
DSC	Differential scanning calorimetry
RH	Relative humidity
SD	Standard deviation
Tg	Glass transition temperature
XRD	X-ray diffractionReferences
