# Supplementary material for: Leucine Protects Dry Powders for Inhalation Against Irreversible Moisture-Induced Aggregation
Source: Pharmaceutics. 2025 Oct 27;17(11):1391. doi: 10.3390/pharmaceutics17111391 (PMC12655236; doi:10.3390/pharmaceutics17111391)
Supplement: Supplementary file 1 [file pharmaceutics-17-01391-s001.zip › pharmaceutics-3905991-supplementary.pdf]

pharmaceutics

MDPI

Article

Leucine Protects Dry Powders for Inhalation Against Irreversible Moisture-Induced Aggregation

Evalyne M. Jansen [1], Luke van der Koog [2,3], Henderik W. Frijlink [1] and Wouter L. J. Hinrichs [1]*

[1] Department of Pharmaceutical Technology and Biopharmacy, Groningen Research Institute of Pharmacy, Faculty of Science and Engineering, University of Groningen, 9713 AV Groningen, The Netherlands; e.m.jansen@rug.nl (E.M.J.); h.w.frijlink@rug.nl (H.W.F.); w.l.j.hinrichs@rug.nl (W.L.J.H)
[2] Department of Molecular Pharmacology, Groningen Research Institute of Pharmacy, Faculty of Science and Engineering, University of Groningen, 9700 AD Groningen, The Netherlands; l.van.der.koog@rug.nl
[3] GRIAC, Groningen Research Institute for Asthma and COPD, University Medical Center Groningen, 9700 RB Groningen, The Netherlands; l.van.der.koog@rug.nl
* Correspondence: w.l.j.hinrichs@rug.nl

Supplementary materials

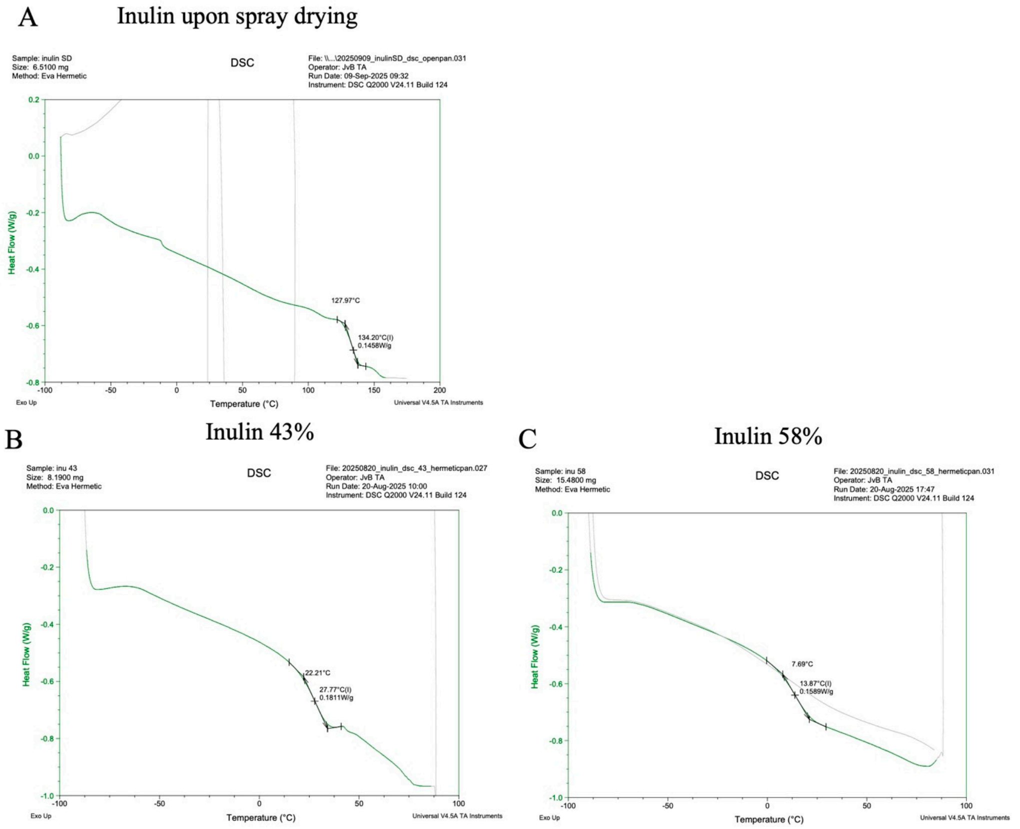

Figure S1. Typical example of DSC measurements of spray dried inulin. (**A**). DSC measurement of spray dried inulin with 4 wt-% leucine directly after spray drying, (**B**). Spray dried inulin 4 wt-% leucine after 1 day of storage at 43% RH, (**C**). Spray dried inulin 4 wt-% leucine after 1 day of storage at 58% RH. *N* = 3.

 https://doi.org/10.3390/pharmaceutics17111391

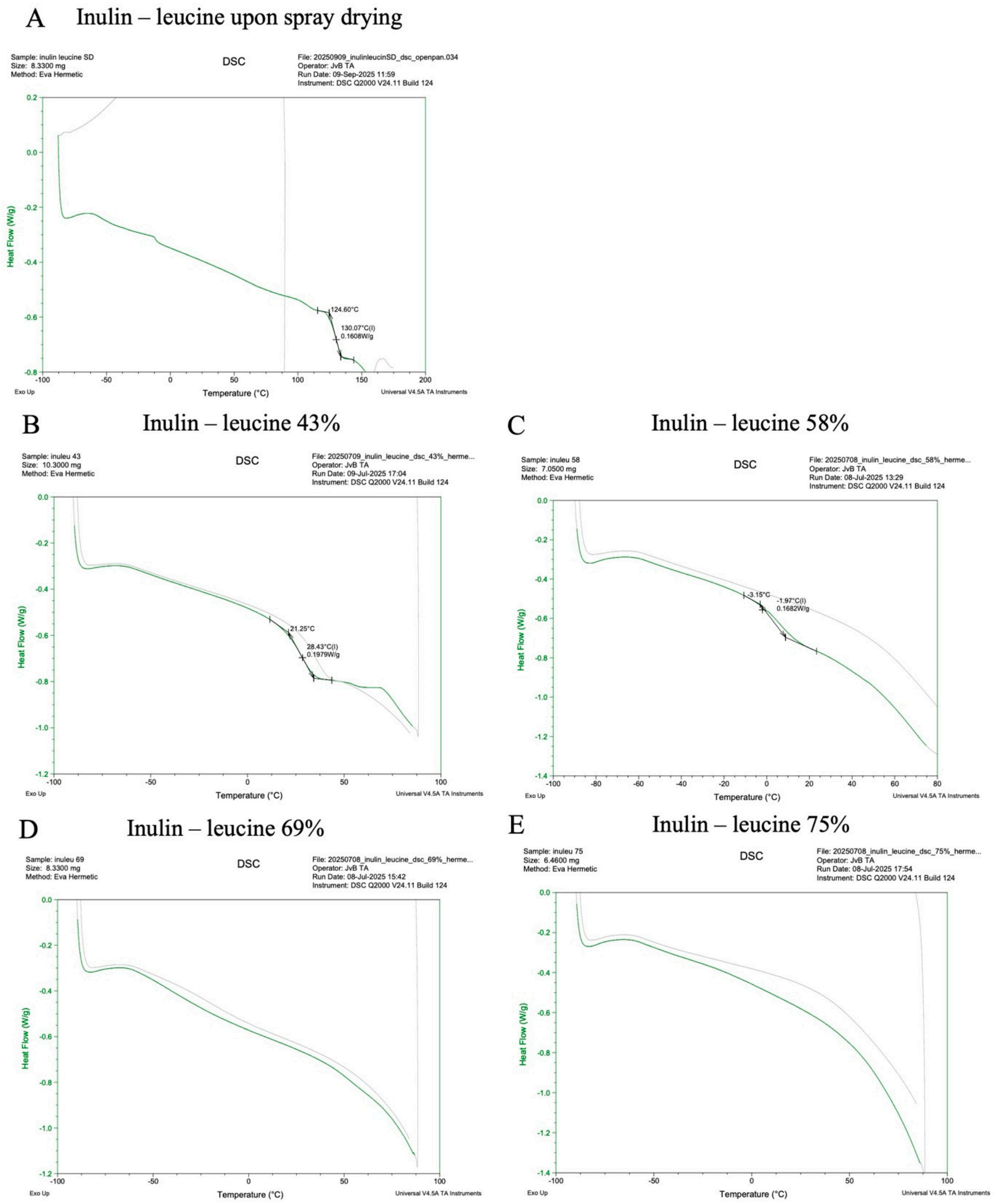

Figure S2. Typical example of DSC measurements of spray dried inulin with 4 wt-% leucine. (**A**). DSC measurement of spray dried inulin with 4 wt-% leucine directly after spray drying, (**B**). Spray dried inulin 4 wt-% leucine after 1 day of storage at 43% RH, (**C**). Spray dried inulin 4 wt-% leucine after 1 day of storage at 58% RH, (**D**). Spray dried inulin 4 wt-% leucine after 1 day of storage at 69% RH, (**E**). Spray dried inulin 4 wt-% leucine after 1 day of storage at 75% RH. $N = 3$.

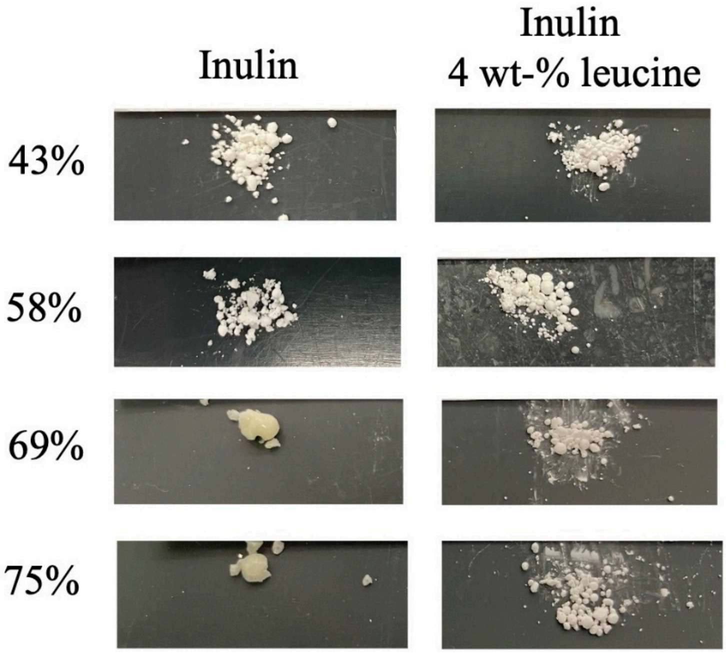

Figure S3. Spray dried inulin and inulin-leucine stored at 43%, 58%, 69% and 75% RH for 1 day. After 1 day of storage spray dried inulin showed viscous flow when stored at 69% and 75% RH, but not for 43% and 58% RH. The inulin-leucine formulation remained a not irreversible aggregated dry powder.

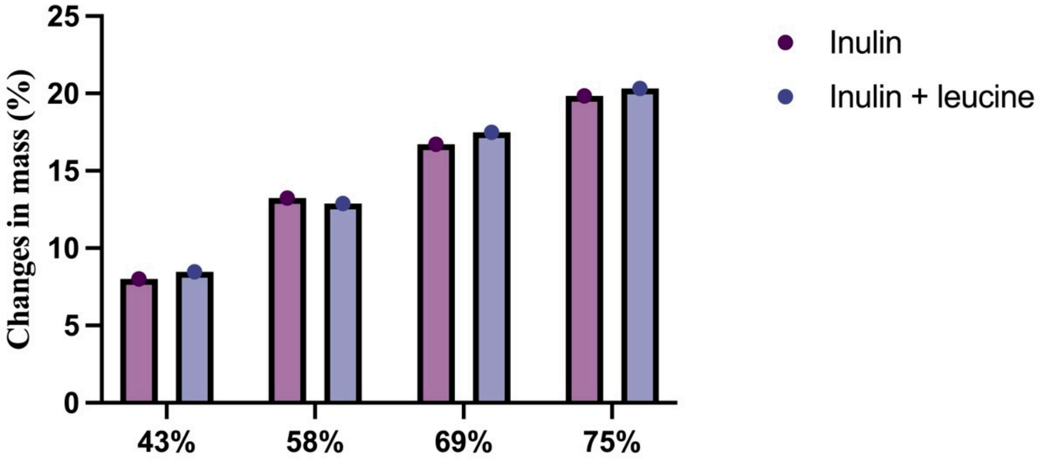

Figure S4. DVS measurement of spray dried inulin and spray dried inulin with 4 wt-% leucine. Change in mass is shown in percentage (%), measured at 43% relative humidity (RH), 58% RH, 69% RH and 75% RH at ambient temperature, *N = 1.*

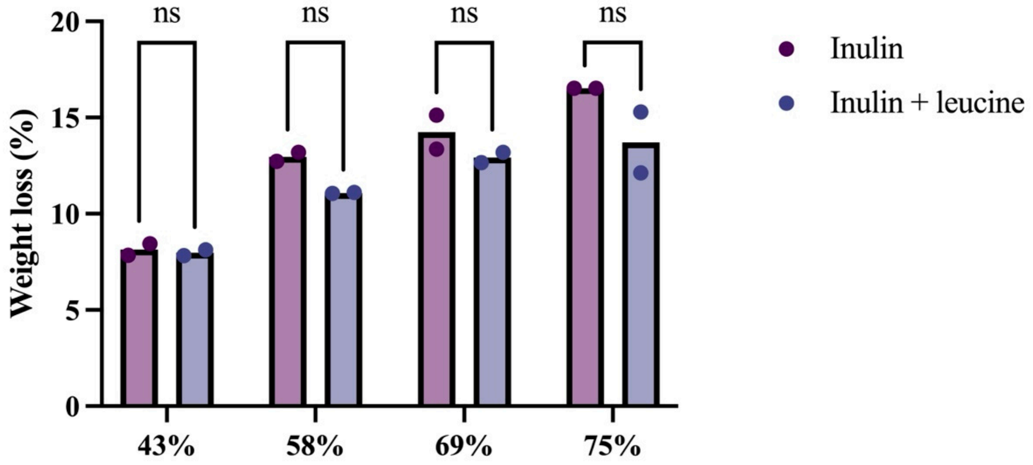

Figure S5. TGA measurement of spray dried inulin and spray dried inulin with 4 wt-% leucine. ns = not significant, 43% relative humidity (RH), 58% RH, 69% RH and 75% RH, *N* = 2.

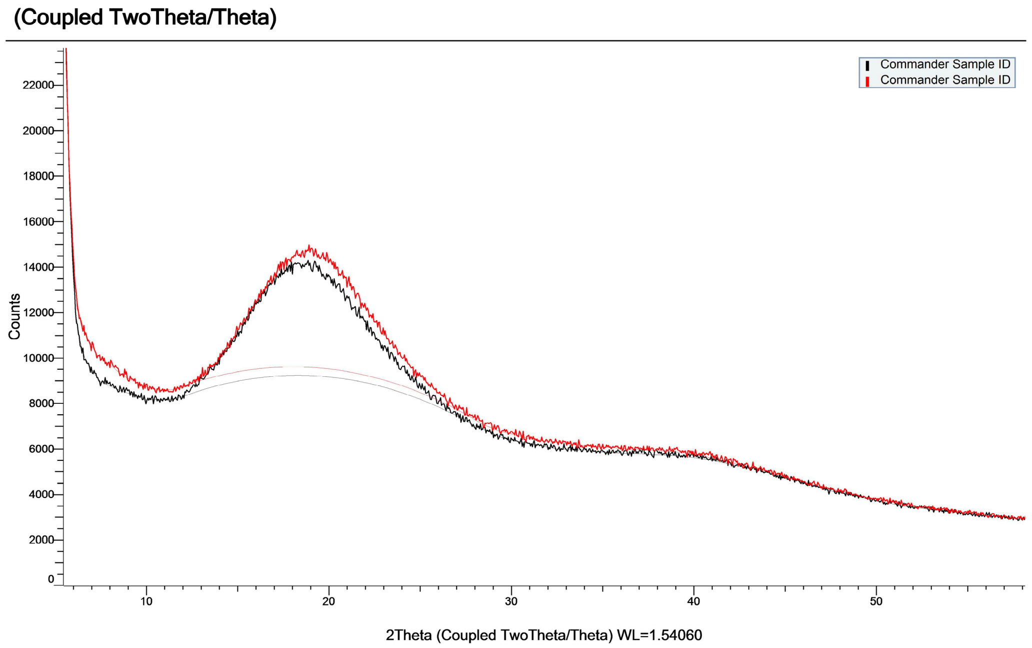

Figure S6. XRPD analysis showed the amorphous structure of spray dried inulin and inulin-leucine formulations. Black = spray dried inulin, red = spray dried inulin-leucine.
